# Effects of Tocilizumab on Adults With COVID-19 Pneumonia: A Meta-Analysis

**DOI:** 10.3389/fmed.2022.838904

**Published:** 2022-03-30

**Authors:** Chi-Chung Chen, Yu-Pei Yang, Hsien-Lung Tsai, Tao-Hsin Tung

**Affiliations:** ^1^Department of Emergency Medicine, Cheng Hsin General Hospital, Taipei, Taiwan; ^2^Department of Hematology, Taizhou Hospital of Zhejiang Province Affiliated to Wenzhou Medical University, Linhai, China; ^3^Enze Medical Research Center, Affiliated Taizhou Hospital of Wenzhou Medical College, Taizhou, China

**Keywords:** COVID-19, tocilizumab, meta-analysis, mortality, side effects, treatment

## Abstract

**Background:**

Coronavirus disease-2019 (COVID-19), a worldwide disaster, has already affected lots of people. Effective care and therapy are currently being evaluated in full swing.

**Purpose:**

Our purpose was to investigate the effects of tocilizumab, an interleukin-6 receptor inhibitor, on treatment of adult patients with COVID-19 pneumonia.

**Data Sources, Study Selection, and Data Extraction:**

We conducted a meta-analysis and searched for relevant studies on Pubmed, Embase, and the Cochrane Library without restrictions on language from inception until February 1, 2021. Fifteen studies were included for this meta-analysis. Two authors independently selected and screened these studies, assessed the quality of included studies, and extracted related information.

**Results:**

Fifteen studies were included in this meta-analysis. The main studies showed that tocilizumab was associated with lower mortality (risk ratio = 0.62, 95% confidence interval = 0.46–0.83; and hazard ratio = 0.61, 95% confidence interval = 0.51–0.72). Using tocilizumab might also affect biochemistry indicators (lowered C-reactive protein and ferritin, increased lymphocyte count).

**Conclusion:**

These current bodies of evidence could indicate that early use of tocilizumab was associated with lower mortality in adult patients with COVID-19. Early use of tocilizumab could reduce the mortality rate of adult patients with COVID-19 without obvious fatal side effects, which may be a treatment option in patients with COVID-19 pneumonia.

**Systematic Review Registration:**

The study protocol was registered on PROSPERO (ID:242811).

## Introduction

An outbreak of pneumonia caused by severe acute respiratory syndrome coronavirus 2 [coronavirus disease 2019 (COVID-19)] emerged in Wuhan, Hubei province, China in December 2019. COVID-19 represents lots of clinical manifestations that typically include fever, cough, and fatigue (1). There is no definite, quite effective treatment for patients with COVID-19 infection until February 2021. Compared to disease caused by previously known human CoVs, COVID-19 showed less severe pathogenesis but higher transmission competence. Since there are no definite antiviral drugs existing to fully treat or prevent COVID-19, potential therapeutic strategies arecurrently being evaluated in full swing (2). For example, systemic steroids have been reported to benefit severely ill mechanically ventilated patients with COVID-19 (3). Severe dysregulated systemic inflammation is the putative mechanism. Thus, early administration of prolonged, low-dose methylprednisolone treatment was associated with lower mortality and decreased ventilator dependence (4).

Tocilizumab (trade name Actemra), also known as atlizumab, is an immunosuppressive drug mainly used for treatment of rheumatoid arthritis and systemic juvenile idiopathic arthritis, a severe form of arthritis in children. This interleukin-6 receptor inhibitor showed possible efficacy and safety in patients with COVID-19. After some review of related literature, we could speculate that using the IL-6 inhibitor tocilizumab might lower the mortality of patients suffering from COVID-19 pneumonia (5–7). However, more substantial and integrative results are required. Because of highly infectious COVID-19 pneumonia, finding out a definite therapy was the top priority. Critically ill patients with COVID-19 might add heavy pressure on the medical system (3). The use of the IL-6 inhibitor in treating COVID-19 is still controversial. The object of this meta-analysis was to assess the curative effect of tocilizumab on COVID-19, regardless of race (including Europeans, Americans, and Asians) and severity of the disease.

## Methods

We performed a meta-analysis of observational studies to examine the effects of tocilizumab on COVD-19. The study protocol was registered on PROSPERO.

### Searching for Literature

We followed MOOSE (Meta-analysis of Observational Studies in Epidemiology) guidelines to conduct and report this study (8). We searched PubMed, Embase, and the Cochrane Library for relevant studies with no language limitation from inception to February 1, 2021. The search strategy is illustrated in Table 1.

**Table 1:** Search strategy in three databases until February 1, 2021.

PubMed	#1 COVID-19: 38175
	#2 2019-nCoV: 16400
	#3 Coronavirus: 41949
	#4 Tocilizumab: 3465
	#5 ACTEMRA: 3467
	#6 (#1 OR #2 OR #3) AND (#4 OR #5): 325
	#7 Filters: Humans: 162
Cochrane Library	#1 Tocilizumab: 1094
	#2 ACTEMRA: 118
	#3 Coronavirus: 974
	#4 COVID-19: 1565
	#5 (#1 OR #2) AND (#3 OR #4):
	52 (1review, 1 protocol, 50 trials)
Embase	#1 Coronavirus: 57501
	#2 Covid-19: 38092
	#3 2091-nCoV: 985
	#4 (#1 OR #2 OR #3): 58547
	#5 ACTEMRA: 551
	#6 Tocilizumab: 12992
	#7 (#5 OR #6):12998
	#8 Filters: Humans: 23264340
	#9 (#4 AND #7 AND #8): 784

### Study Selection

We included observational studies that examined the effects of tocilizumab on COVID-19. Two authors (Chi-Chung Chen and Hsien-Lung Tsai) independently scanned the search results, reviewed the full text of potential studies, and decided whether these studies met our selection criteria. The third author (Tao-Hsin Tung) was consulted if there was any disagreement.

### Data Extraction and Risk of Bias Assessment

The following data were extracted from the included studies: first author, year of publication, country, study subjects, and outcomes. Outcomes included mortality rates between tocilizumab and control groups. At the end of 2020, treatment of COVID-19 was various. Classes of drugs used are antiviral agents (e.g., lopinavir/ritonavir and remdesivir), inflammation inhibitors/antirheumatic drugs (e.g., tocilizumab, antimalarials, and methylprednisolone), low molecular-weight heparin, plasma, and hyperimmune immunoglobulins (3, 9). Definite therapy was still under development and research. Two authors (Chi-Chung Chen and Hsien-Lung Tsai) independently utilized the Newcastle-Ottawa Scale (NOS) to assess the quality of included non-randomized studies. The NOS applies three domains (selection of study groups, comparability, and outcome assessment) to evaluate the quality of these studies (10). A study could be awarded up to one star for each item in the selection and outcome domains and up to two stars for comparability. We considered a study of high quality if seven or more stars were awarded. Any disagreement was adjudicated by the third author (Tao-Hsin Tung).

### Statistical Analysis

Review Manager 5.4.1 (Copenhagen: The Nordic Cochrane Center, The Cochrane Collaboration, 2020) was used to conduct the meta-analysis. We presented the risk of outcome as risk ratio (RR), hazard ratio (HR), and 95% confidence interval (CI), and assessed heterogeneity using I^2^ statistic, which evaluated the degree of variation across studies that was due to heterogeneity rather than chance alone. An I^2^ value of ≥50% represented substantial heterogeneity. We conducted a random-effects model meta-analysis because we expected considerable clinical heterogeneity.

## Results

### Characteristics of Included Studies

**Figure 1:**
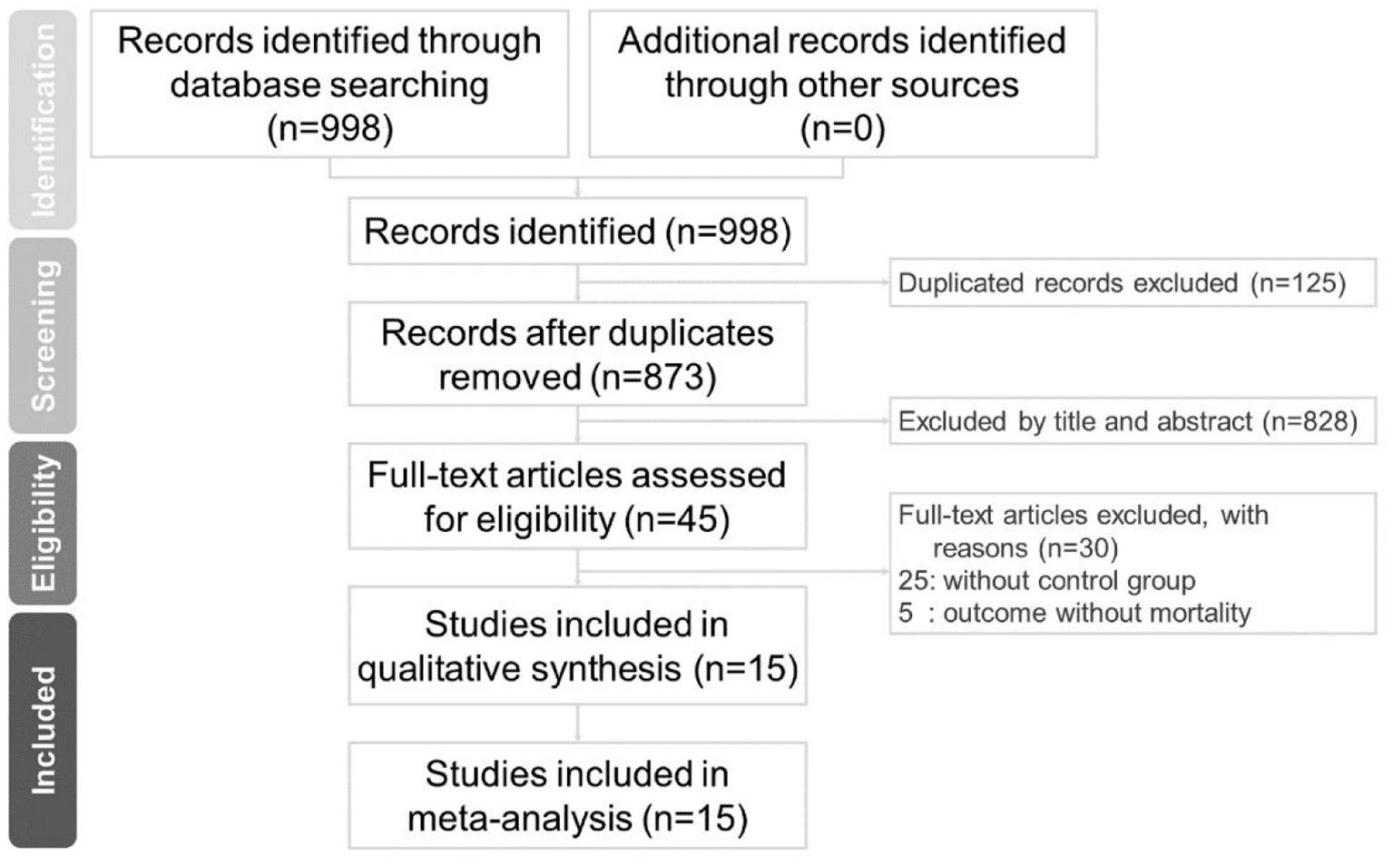
Preferred Reporting Items for Systematic and Meta-Analyses (PRISMA) study flow chart.

There were 784 studies found on Embase, 142 on PubMed. and 52 studies on the Cochrane Library. After removing duplicates, 853 articles were identified. A total of 15 studies meet the inclusion criteria, as shown in Figure 1. A total of 1,993 study subjects were involved in these 15 studies. Table 2 illustrates the characteristics of these included studies. Most of the included studies were conducted in Europe and the United States. All the included studies were focused on the effects of tocilizumab on the severity of COVID-19, and all the studies contained experiment groups (patients treated with tocilizumab) and control groups (patients not treated with tocilizumab).

**Table 2:** Characteristics of included studies.

**First author /Year/Country**	**Database used/duration**	**Inclusion criteria**	**Study subjects**	**Primary outcome**	**NOS score**	**Outcome:mortality**
Campochiaro et al. (11) 2020, Italy	San Raffaele Hospital 19 March	Patients with COVID-19 pneumonia	32 treated with TCZ, 33 matched served as the control group	28-day follow up	S**** C** O***	TCZ treated: 15% vs. Untreated 33%, *P* = 0.15
Somers et al. (12) 2020, USA	Michigan Medicine 9 March and 19 May 2020	Aged >16 years with severe COVID-19 pneumonia, mechanically ventilated	154 requiring mechanical ventilation: 78 treated with TCZ and 76 untreated	survival probability postintubation	S **** C ** O ***	0.55 (0.33–0.90) 14-day: TCZ treated 9% VS Untreated 26%, *P* = 0.005 21-day: TCZ treated 14% vs. Untreated 25%, *P* = 0.006 28-day: TCZ treated 18% vs. Untreated 36%, *P* = 0.01
Guaraldi et al. (13) 2020, Italy	Azienda Ospedaliero-UniversitariaPoliclinico of Modena, Azienda USL-IRCCS di Reggio Emilia, Reggio nell'Emilia and tertiary care centers in Bologna 21 Febrary and 30 April 2020	Aged ≥ 18 years with severe COVID-19 pneumonia	179 treated with TCZ (91 with subcutaneous TCZ, 88 with intravenous TCZ), 365 matched served as the control group	invasive mechanical ventilation or death	S**** C** O***	TCZ treated: 7% (subcutaneous: 8%, intravenous: 7%) vs. Untreated: 20%, *P* = 0.0007
Rojas-Marte et al. (14) 2020, USA	Maimonides Medical Center 8 March and 25 April 2020	adult patietns with severe to critical COVID-19 pneumonia	96 treated with TCZ, 97 matched served as the control group	overall mortality rate	S**** C** O**	TCZ treated 44.8% vs. Untreated 56.7%, *P* = 0.09 Excluding intubated patients: TCZ treated 6% vs. Untreated 27%, *P* = 0.024
Hill et al. (15) 2020, USA	University of Washing Enterprise Data Warehouse 3 March and 24 April 2020	Patients with COVID-19 pneumonia	43 treated with TCZ, 45 matched served as the control group	28-day follow up	S**** C** O**	TCZ treated: 20% vs. untreated: 33 0.26 (0.21–1.52)
Eimer et al. (16) 2020, Sweden	Karolinska University Hospital Huddinge 11 March and 15 April 2020	Aged ≥ 18 years with COVID-19 pneumonia	29 treated with TCZ, 58 matched served as the control group	30-day all-cause mortality after admission to ICU (=day 0)	S**** C** O***	30-day: TCZ treated: 17.2% vs. Untreated 32.8%, 0.52 (0.19–1.39), *P* = 0.19
Patel et al. (17) 2020, Sweden	Swedish Medical Center 16 March and 17 April 2020	Aged ≥ 18 years with COVID-19 pneumonia	42 treated with TCZ (21 severe, 21critical), 41 matched served as the control group	7 days of follow-up data or discharged/died before day 7	S**** C** O**	7-day: TCZ treated severe 14.2%/TCZ treated critical 28.6% vs. Untreated 28.6%,
Alberrtini et al. (18) 2020, France	Le Raincy-Montfermeil Hospital Center 6 April and 21 April 2020	Patients with COVID-19 pneumonia	22 treated with TCZ, 22 matched served as the control group	14 days of follow-up or mortality	S**** C** O**	TCZ treated: 13.6% vs. 9.1%
Canziani et al. (19) 2020, Italy	Humanitas Gavazzeni Bergamo and Humanitas Milan 15 March and 22 April 2020	adult patients with COVID-19 pneumonia, need of respiratory support	64 treated with TCZ, 64 matched served as the control group	death	S**** C** O***	30-day: TCZ treated: 27% vs. Untreated: 38%, 0.61 (0.33–1.15)
Potere et al. (20) 2020, Italy	Pescara General Hospital 28 March and 21 April 2020	patients with COVID-19 pneumonia	40 treated with TCZ+SOC, 40 treated with SOC	discharge or death (observe 35 days)	S**** C** O***	14-day: TCZ treated 2.5% vs. Untreated 12.5%, *P* = 0.003 < survival free of invasive mechanical ventilation> 35-day: TCZ treated 5.0% vs. Untreated 27.5%, *P* = 0.006 < mortality>
Moreno-Pérez et al. (21) 2020, Spain	Academic Spanish hospital 12 March and 2 May 2020	patients with COVID-19 pneumonia	77 treated with TCZ, 159 matched served as the control group	discharge alive from ICU by day 14	S**** C** O***	TCZ treated: 12.9% vs. Untreated 1.9%, *P* = 0.0001
Capra et al. (22) 2020, Italy	Montichiari hospital 26 February and 2 April	patients with COVID-19 pneumonia and respiratory failure, not needing mechanical ventilation	62 treated with TCZ, 23 matched served as the control group	survival rate in patients treated with tocilizumab and controls	S**** C** O***	TCZ treated: 3.2% vs. Untreated: 47.8%, 0.035 (0.004–0.347), *P* = 0.004
Rossotti et al. (23) 2020, Italy	ASST Grande Ospedale Metropolitano Niguarda 13 March and 3 April 2020	Aged ≥ 18 years with severe/critical COVID-19 pneumonia	74 treated with TCZ, 148 matched controls with standard of care	overall mortality	S**** C** O**	TCZ vs. Untreated 0.499 (0.262–0.952), *P* = 0.035
T. Klopfenstein et al. (24) 2020, France	Nord Franche-Comté Hospital 1 March and 24 April 2020	patients with COVID-19 pneumonia	20 treated with TCZ, 25 matched served as the control group	death and/or ICU admissions	S**** C** O***	TCZ treated: 25% vs. Untreated 72%, *P* = 0.002
Gokhale et al. (25) 2020, India	Lokmanya Tilak Municipal Medical College and General Hospital 20 April and 5 June	patients with COVID-19 pneumonia	70 treated with TCZ, 91 matched served as the control group	death	S**** C** O***	TCZ treated: 47.1% vs. Untreated 67%, *P* = 0.011

*Each asterisk means one star, and the total scale of NOS is the summation of the stars (nine is the maximum)*.

Drugs used in control groups were various, such as hydroxychloroquine, azithromycin, remdesivir, and methylprednisolone (12, 14, 23). It is worth acknowledging that using adjunctive corticosteroid in patient with COVID-19 with acute respiratory distress syndrome could be a choice (12, 26).

The risk of bias assessment for included studies is also illustrated in Table 2. We used The Newcastle-Ottawa Scale (NOS) to assess the quality of these studies (10). Despite the short research time, there were two studies considered to not have a relatively long study period (<2 weeks) (8, 9). However, these two studies still had some valuable statistical information.

### Benefits of Using Tocilizumab in Patients With COVID-19

**Figure 2:**
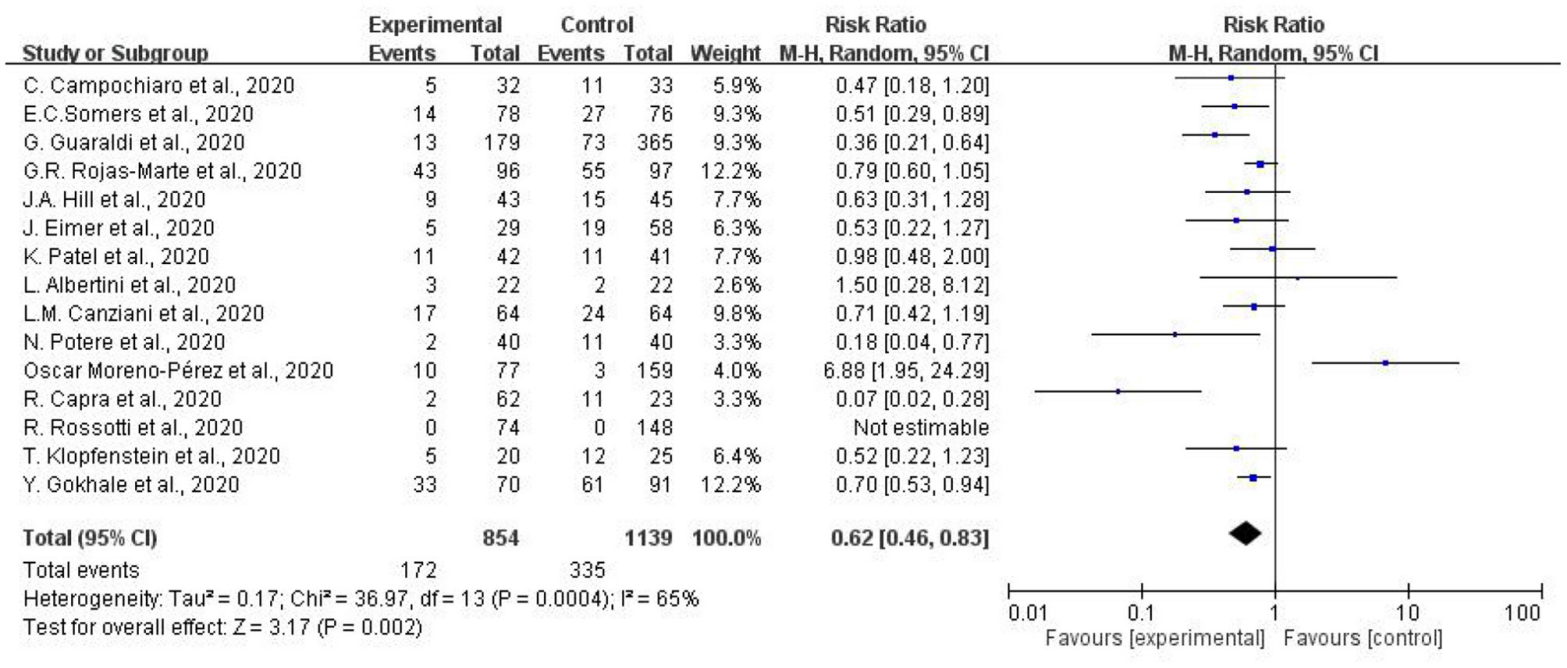
Meta-analysis of effects of tocilizumab on coronavirus disease-2019 (COVID-19) (risk ratio).

Figure 2 indicates the results of mortality in patients with COVID-19 receiving tocilizumab. Fifteen studies with a total of 854 COVID-19 patients receiving tocilizumab and 1139 patients as controlled group were collected. Our study found substantial heterogeneity (I^2^ = 65%) among the 15 studies. Overall decreased mortality was found (pooled RR:0.62, 95% CI = 0.46–0.83). Subgroup heterogeneity was significant (I^2^ = 65%).

**Figure 3:**
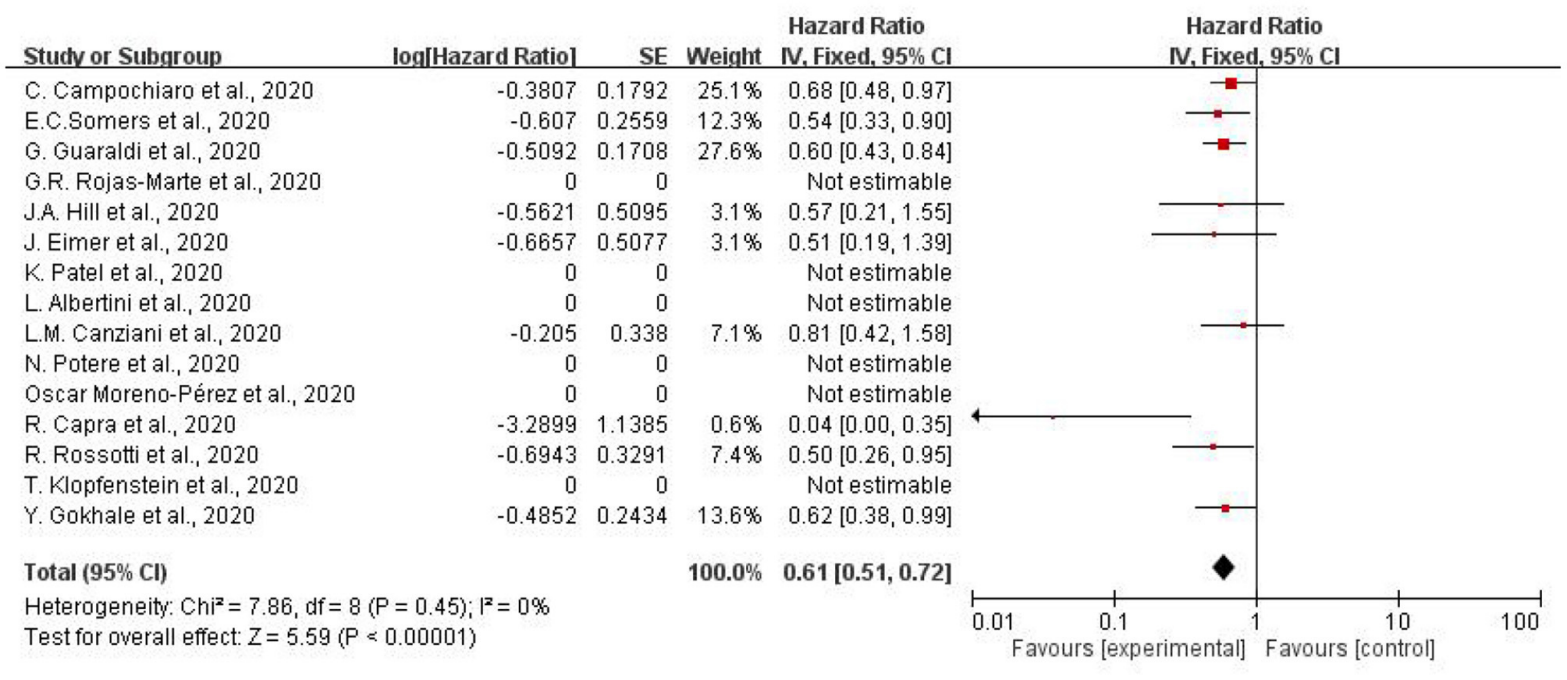
Meta-analysis of effects of tocilizumab on COVID-19 (hazard ratio).

Figure 3 illustrates the hazard ratio of using tocilizumab to treat patients with COVID-19. Overall decreased mortality was also found (pooled HR.61, 95% CI = 0.51–0.72). I^2^ of subgroup heterogeneity was 0%. In summary, adult patients suffering from COVID-19 showed lower mortality compared to the controlled-group, which was treated with other therapy.

### Good Effect of Using Tocilizumab

Among our studies, one of them showed a good effect of using tocilizumab on treatment of patients with COVID-19. Patients receiving tocilizumab showed significantly better survival rate than control groups (HR for death 0.035; 95% CI =0.004–0.347; *p* = 0.004) (22). After detailed analysis, the included subjects were all diagnosed as having COVID-19 but without mechanical ventilation. Maybe the relatively less severity of COVID-19 infection could explain the good effect of using tocilizumab.

### Disadvantages of Using Tocilizumab in Patients With COVID-19

Tocilizumab was associated with improved survival; however, it increased the proportion of patients with superinfections (54 vs. 26%; *p* < 0.001). Despite higher superinfection occurrence, there was no difference in 28-day case fatality rate (12).

Possibly because of the result of several biochemical, respiratory, and infectious complications, using tocilizumab in patients with COVID-19 was associated with longer hospital stay (HR 1.658, 95% CI = 1.088–2.524, *p* = 0.019) (23). By the way, a study in India showed longer days of hospitalization in the tocilizumab group (14 days) than in the control group (6 days) (*p* = 0.001) (25).

On the contrary, another study conducted in Sweden showed different results. Median length of stay in hospital was 20.5 (days) in the tocilizumab group and 30 (days) in the control group (*p* = 0.04) (16).

### Variety of Biochemistry Indicators After Using Tocilizumab in Patients With COVID-19

Treating COVID-19 with tocilizumab showed some variations in biochemistry indicators. After treatment, C-reactive protein (CRP) showed a clear reduction (*P* < 0.001). Meanwhile, D-dimer and lymphocyte both showed a significant increase (*p* < 0.001) (23). Despite short period, tocilizumab still showed positive influence on lowering CRP level (from 23.7 to 0.9 mg/dl) and ferritin level (from 1,522 to 940 ng/ml) and increasing lymphocyte count (from 800 to 1,700 cell/mmc) (17). Although these acute phase reactants are unreliable biomarkers for disease improvement, changes in biochemistry indicators could help us know about patients' current physical condition.

### Corticosteroid in the Study

In addition to tocilizumab, a corticosteroid was used in eight of the fifteen studies (among both experiment and control groups). Since steroids had been reported to benefit patients with COVID-19 with acute respiratory distress syndrome (26), use of steroids had no influence on results of the studies (12, 14, 16, 19).

### Publication Bias of the Study

**Figure 4:**
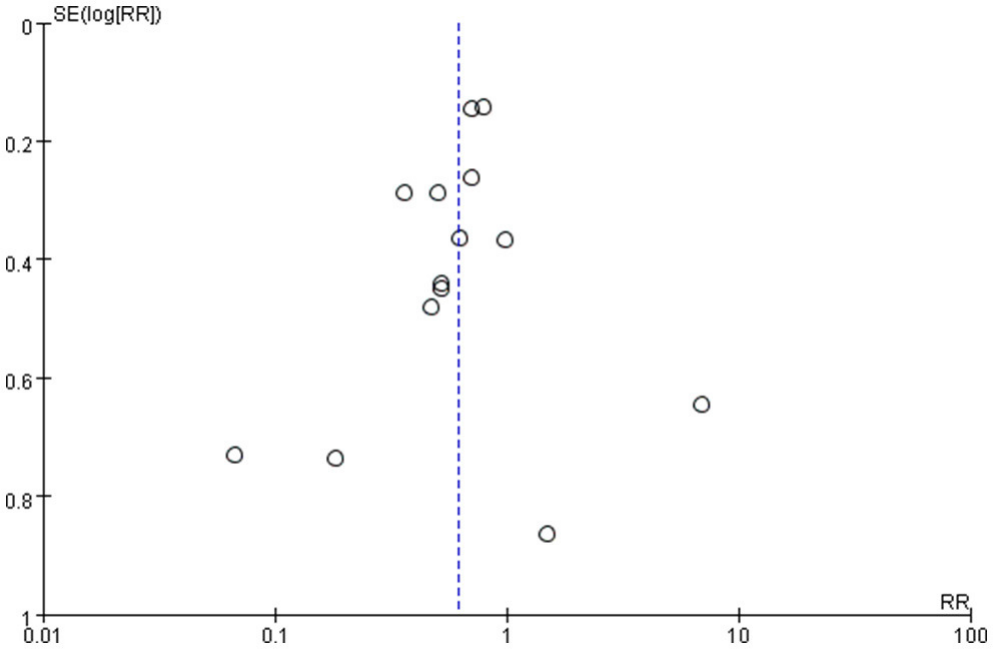
Bias assessment plot (risk ratio).

**Figure 5:**
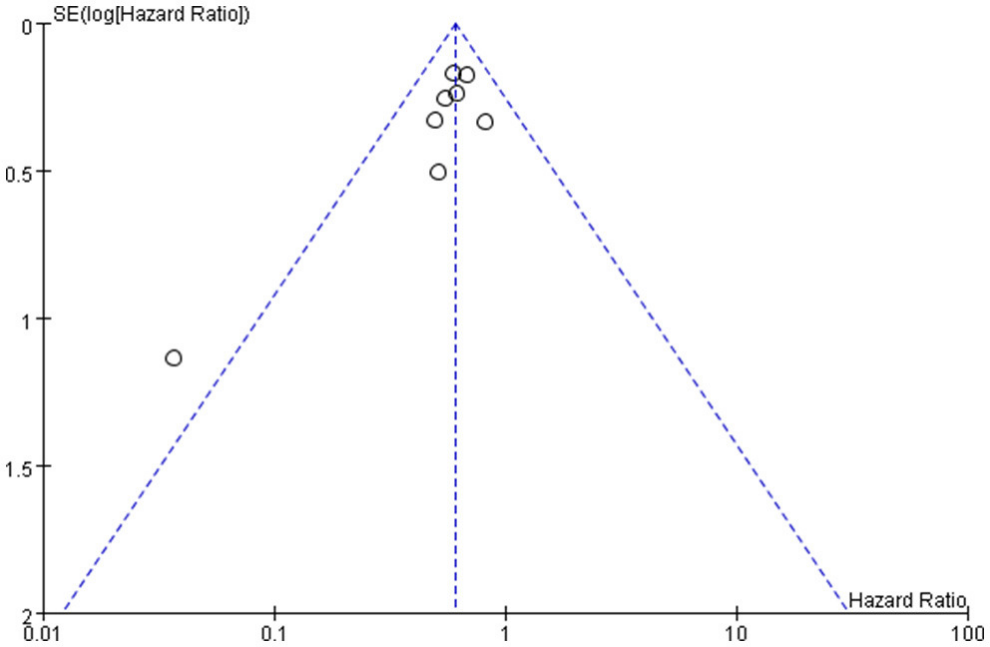
Bias assessment plot (hazard ratio).

Publication bias was defined as publication or non-publication of studies depending on the direction and statistical significance of the results. Figures 4, 5 show that the funnel plots are not absolutely symmetry, which indicates some inevitable publication bias in this study.

## Discussion

### Clinical Implications

In this meta-analysis of fifteen studies, as of February 2021, we observed that tocilizumab reduced the mortality of adult patients with COVID-19 pneumonia infection. The results indicated that early use of tocilizumab was associated with lower mortality in adults with COVID-19 pneumonia. Perhaps the relative less severity of COVID-19 infection could explain the good effect of using toxilizumab in treatment of patients with COVID-19. Lots of patients with COVID-19 suffered from severe complications, such as respiratory distress, intensive care unit (ICU) stay and even death. Current studies suggest that increased alveolar exudates caused by host immune response and inflammatory cytokine storm might contribute to mortality (27).

As observed in respiratory distress syndrome, interleukin-6 (IL-6) played a key role in inducing acute inflammation. The recombinant human monoclonal antibody tocilizumab, directed against the IL-6 receptor, mediated an inhibition strategy, preventing IL-6 binding to its receptor and, therefore, IL-6 biological activity (6).

The tocilizumab treatment showed inspiring clinical results (27). Tocilizumab is a potential recombinant monoclonal antibody against IL-6. Currently, tocilizumab is under investigation for management of patients with COVID-19 infection (28).

Previous studies have assessed the efficacy of tocilizumab in treatment of COVID-19 and showed that all-cause mortality of COVID-19 between tocilizumab and control groups was 16.3 and 24.1%, respectively. However, the difference did not have statistical significance (95% CI = 0.31–1.22). Besides, a similar risk of ICU admission was noted between the tocilizumab and control groups (35.1 vs. 15.8%, 95% CI = 0.33–6.78). From a clinical viewpoint, no clear evidence showed that tocilizumab could provide definite benefit to patients with COVID-19 infection (29).

From an evidence-based medicine viewpoint, ordinary meta-analyses on the efficacy of interventions obtain confident quality evidence from randomized controlled trials only (30–32). However, randomized trials are often not the best source of clinical evidence on harm, as the period of study is often too short to detect long-term or rare adverse outcomes (33, 34). Including observational studies in our analysis was a strength, as these studies could detect the effect of tocilizumab, which might be important in treatment of patients with COVID-19 pneumonia.

### Methodological Considerations

Another strength of our study was that we included most case-control studies regarding the possible therapeutic effects of tocilizumab on treatment of patients with COVID-19 pneumonia.

Several limitations should be considered when interpreting the results of this meta-analysis. First, the main limitation of the study was inclusion of only observational studies. There were not enough double-blind randomized controlled trials until February 2021. Second, research time was not long enough. Due to this urgent global crisis, nearly all studies to date have a relatively short research time. The longest research time is only more than a few months. Third, the main research subjects included in our studies were adults. Effect of the treatment on minors was still unknown. Lastly, the patients enrolled in the study were treated with different drugs and started treatment with tocilizumab in different stages of infection. Disease severity was inconsistent.

### Heterogeneity of Meta-Analysis

In this meta-analysis, heterogeneity may exist if sample estimates for the population risk were of different magnitudes. For existence of heterogeneity, it is crucial to assess heterogeneity in the meta-analysis. We aggregate studies that have different methodologies; however, heterogeneity in the results is still inevitable.

## Conclusion

This meta-analysis included 15 publications with more than 1,993 individuals to analyze the correlation of tocilizumab with clinical mortality among adult patients with COVID-19 pneumonia. Although the patients in our study were treated with tocilizumab in different stages of disease, our study showed that early use of tocilizumab was associated with lower mortality in adults with COVID-19 pneumonia. Despite some possible increased risks of infection and prolonged hospital stay, the use of tocilizumab could reduce mortality without obvious fatal side effects, which might be a treatment option for adult patients with COVID-19 pneumonia if it is used early. Besides, relatively less severe symptoms of COVID-19 pneumonia might explain the good effect of using tocilizumab.

Further studies should have a more in-depth insight into the effect of tocilizumab on treatment of patients with COVID-19 pneumonia.

## Data Availability Statement

The original contributions presented in the study are included in the article/supplementary materials, further inquiries can be directed to the corresponding authors.

## Author Contributions

C-CC and H-LT conducted the study and drafted the manuscript. T-HT and Y-PY participated in the design and data collection of the study, conceived the study, and participated in its design and coordination. C-CC and T-HT calculated the results of this study. All the authors read and approved the final version of the manuscript.

## Conflict of Interest

The authors declare that the research was conducted in the absence of any commercial or financial relationships that could be construed as a potential conflict of interest.

## Publisher's Note

All claims expressed in this article are solely those of the authors and do not necessarily represent those of their affiliated organizations, or those of the publisher, the editors and the reviewers. Any product that may be evaluated in this article, or claim that may be made by its manufacturer, is not guaranteed or endorsed by the publisher.
